# Spatial patterns and predictors of unintended pregnancy among reproductive age women in Ethiopia

**DOI:** 10.1371/journal.pone.0282225

**Published:** 2023-08-02

**Authors:** Melkamu A. Zeru, Haile Mekonnen Fenta, Aweke A. Mitku

**Affiliations:** 1 Department of Statistics, College of Science, Bahir Dar University, Bahir Dar, Ethiopia; 2 School of Mathematics, Statistics and Computer Science, College of Agriculture Engineering and Science, University of KwaZulu-Natal, Durban, South Africa; Addis Ababa University School of Public Health, ETHIOPIA

## Abstract

**Introduction:**

Unintended pregnancy is amajor sexual and reproductive health problem that imposes substantial health, economical and psychosocial costs to individuals and society as well as significant emotional distress to women, families, and society. The main aim of this study was to investigate the spatial distribution and predictors of unintended pregnancy in Ethiopian regions and administrative zones.

**Methods:**

This study was conducted based on data from 2016 Ethiopian Demographic and Health Survey. The prevalence of unintended pregnancy across regions and or zones was assessed using spatial analysis, and the effect of different factors on unintended pregnancy in Ethiopia was investigated using a generalized linear mixed model with a multistage clustered sampling strategy. The crude and best linear unbiased predictor estimations of zones were integrated with the shape file data to demonstrate the performance of each zone on maps.

**Results:**

The prevalence of unintended pregnancy for reproductive women in Ethiopia was29.49%. The highest rates of unintended pregnancy were recorded in the North Gondar zone of the Amhara region and the Jima zone in the Oromiya region. The mixed effects model revealed that age [AOR = 0.78, 95% CI, 0.62–0.97], residence [AOR = 2.62, 95%CI, 1.94, 7.27], marital status [AOR = 0.05, 95%CI, 0.01–0.38], women education [AOR = 1.34, 95%CI, 0.75–2.39], smoking cigarettes [AOR = 3.67, 95CI, 1.17–11.56], and poorer wealth index [AOR = 1.89, 95% CI, 1.51–2.31] were significantly associated with unintended pregnancy.

**Conclusion:**

In Ethiopia, unintended pregnancy is a public health issue, and prevention stratagem for unintended pregnancy among reproductive women need to be focused based on the identified predictors. The spatial distribution of unintended pregnancy varied greatly at zonal and regional levels in Ethiopia. Hence, we recommended that, creating awareness of sexual and reproductive health with special priority to the identified hotspot areas (Amhara, Oromiya and SNN regions) to reduce unintended pregnancy. Emphasis on fertility and contraceptive techniques should be given to couples by health professionals.

## Introduction

Unintended pregnancies are pregnancies that are mistimed, unplanned or unwanted at the time of conception. Unintended pregnancy is a major sexual and reproductive health problem that imposes substantial health, economical and psychosocial costs to individuals and society as well as significant emotional distress to women, families, and society [1].

According to global statistics annually 19 million women in developing countries and more than 15 million in Asia experience unsafe abortion. According to the United Nations World population prospects (2022), the current fertility rate for Ethiopia in 2022 is 3.918 births per woman. The Ethiopian government has made a massive expansion of health facilities and trained human support capacity. Accordingly, the contraceptive prevalence rate (CPR) of the country has increased from 8% in 2000 to 41.4% in 2019 [2].

Sub-Saharan African countries are also suffering from unintended pregnancy [3]. In most African countries including Ethiopia, abortion remains both unauthorized and unsafe which is a leading cause of maternal death. The rate of unplanned pregnancy was much higher in Eastern Africa than in Northern, Southern and Western Africa, where the rate ranges between 56 and 83 per 1,000 women. The prevalence of unintended pregnancy in Kenya, Egypt, Nigeria, and South Africa, with rates of 24, 30.7, 35.9, and 64.33%, respectively [4–7]. Unintended pregnancy was also found to be high in Ethiopia, with rates ranging from 13.7 to 41.5% [8–10]. In Ethiopia, more than one-third of pregnancies (38%) were unintended in 2014, which was somewhat lower than 42% in 2008. According to the 2016 EDHS, 25% of all births in the previous five years and current pregnancies are unintended. Furthermore, according to the 2016 EDHS study, the overall gap between the desired fertility rate and the total fertility rate is one child, implying that Ethiopian women are currently having one child more than they desire [11].

Different studies in Ethiopia revealed that age, residence, religion, marital status, parity, visiting health professionals, history of abortion, age at first birth, family size, educational status, gravidity, distance from a health facility, history of stillbirth, and knowledge of modern contraceptive methods were all identified as determinants of unintended pregnancy [8–10, 12–14].

Moreover, in Ethiopia based on the geographic diversity of unintended pregnancy and its determining factors, policymakers in Ethiopia can use spatial analysis to prevent unintentional pregnancy [15]. However, no previous studies in Ethiopia have identified the zonal and regional variation with its determinants. Detecting the problem of unintended pregnancy and its variation among administrative zones provides deeper insight into the countries’ health priorities since zone’s health departments have the mandate to plan, follow up, monitor, and evaluation of health activities at the lower level. As the administrative zones are mainly ethnic-based, the assessment of zones provides cultural practices regarding the geographic environment of the community in the zones [16–19].

Tobler’s first law states that everything is related to everything else, but close things are more related than far things [20], which is the heart of spatial autocorrelation statistics and is fundamental to all spatial techniques, including geographic space. As a result, this research aimed at the spatial analytic approach to address the issues where in Ethiopia are the hotspot regions for unintended pregnancy and what are the circumstances that cause such wide-spread unintended pregnancies in Ethiopia. As this will aid in pinpointing regions and zones with high rates of unintended pregnancies and guide decision-making in such hotspot regions.

Regarding various socioeconomic and geographic areas, there is a significant skew in the distribution of unintended pregnancy among reproductive women. However, no spatial analyses have been done to pinpoint locations where reproductive women in Ethiopia are most likely to become pregnant unintentionally. Furthermore, there is still a lack of knowledge in Ethiopia on the prevalence and contributing factors of unintended pregnancy among reproductive women. Thus, the spatial distribution and determinants of unintended pregnancy among Ethiopian women of reproductive age were the focus of this study.

## Methods

### Study design, period and setting

A population-based cross-sectional study was utilized in Ethiopia based on the 2016 EDHSs, a nationally representative survey that took place from January 18 to June 27, 2016, and used a sophisticated sample strategy. A two-stage stratified random sampling procedure was utilized in the survey. After stratifying each region into rural and urban areas, 645enumeration areas (EAs) were chosen. A total of 18,008 households were considered, of which 16,650 households and 15,683 women were eligible. At the time of the survey, 1,073 of these women were pregnant and were included in this study.

**Fig 1:**
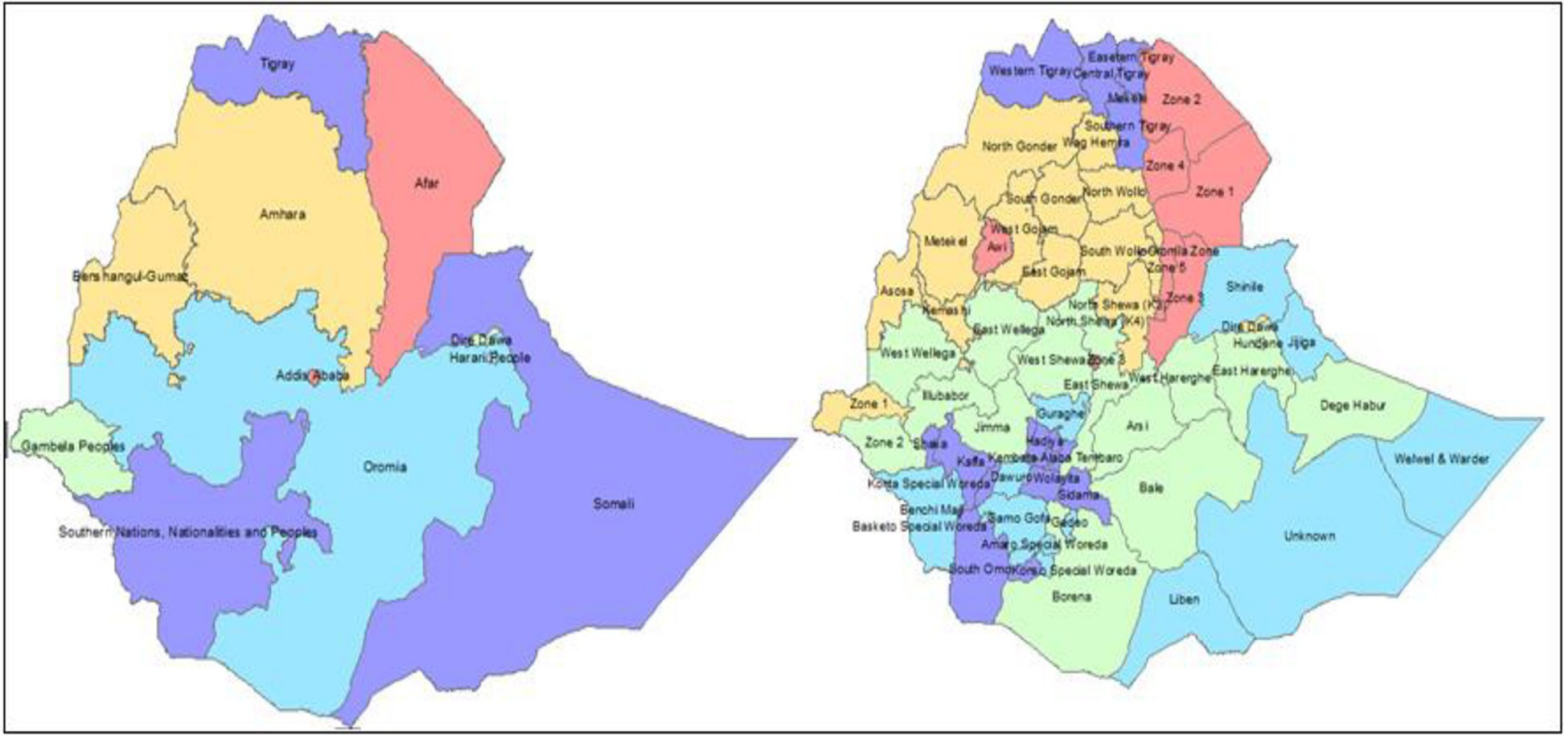
Ethiopian map with 11 regions and 72 administrative zones.

Ethiopia is organized into four administrative levels: region, zone, woreda, and kebele. Ethiopia’s first administrative division is the region, also known as a “kilil” or a regional state. The regions of Ethiopia are defined by ethnolinguistic areas. Currently, in 2022, there are 11 regions (Afar, Amhara, Oromia, Benishangul-Gumuz, Somali, Gambela, Harari, Sidama, Southern, South West, and Tigray) and 2 independently administrative cities (Addis Ababa and Dire Dawa). Zones are created by subdividing regions. Zones are administrative subdivisions in Ethiopia where DHS shifting guarantees that no survey clusters are outside of the zone (Fig 1).

### Data collection procedure and variables

**Fig 2:**
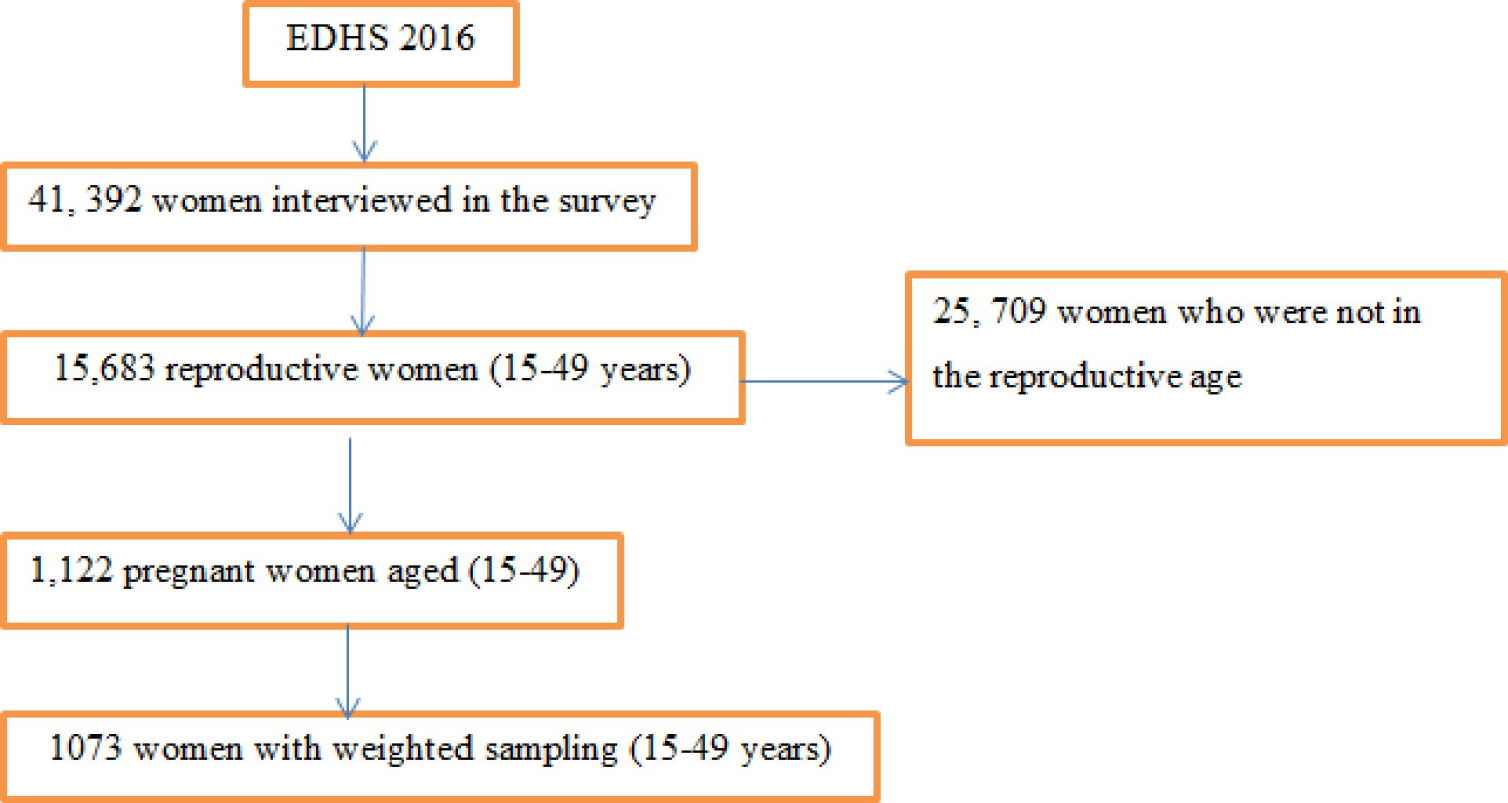
Schematic presentation of inclusion and exclusion criteria for the study samples.

The EDHS 2016 birth information set was utilized and the subordinate variable with its imperative indicators was extricated. The geographic facilitate information was moreover taken from chosen identification clusters. The response variable was unintended pregnancy among women aged 15–49 years in their later pregnancy. The response variable was dichotomized as Yes (coded 1) if women detailed whether their later pregnancy was unintended and No (coded 0) if the pregnancy was wanted. The independent factors were age, religion, wealth index, media access, working status, number of under 5-year children, chewing of khat, smoking cigarettes, education status of husband, current marital status and alcohol use. The inclusion and exclusion criteria’s for this study was considered and it was presented with schematically (Fig 2).

### Data management

Stata 16 was used to extract data, code it, and perform the analysis. To mitigate the influence of sampling error, the data were weighted with the available sample weight factor (v005) within the EDHS dataset. To account for over-sampling, under-sampling, and non-response, the data were weighted.

### Data analysis

The spatial data analysis was utilized to answer the difficult location-based prevalence of unwanted pregnancy and better understand where and what is happening in our research region.

A statistical measure of spatial autocorrelation (Global Moran’s I) was used to assess whether an unintended pregnancy was scattered, clustered, or randomly distributed in the study area. Moran’s I values close to -1 indicated an unintended pregnancy is dispersed among women of reproductive age, while the values close to +1 indicated an unintended pregnancy among women was clustered and the unplanned pregnancy was randomly distributed if the I value was zero [21].

Hotspot analysis was performed by calculating the Gini Index (GI*) statistic for each area and statistical outputs with a high GI* indicate hotspot area, while outputs with a low GI* indicate cold spot areas. The GI* statistic is a z-score in which a high z-score and a small p-value for a feature indicate a significant hotspot, and a low negative z-score with a small p-value indicates a significant cold spot. This analysis was used to identify statistically significant spatial clusters with high (hot spot) and low values (cold spot) of unintended pregnancy among reproductive women in Ethiopia.

The response variables are considered to be independent with density functions belonging to the exponential family based on a vector of individual random effects u_i_. The response variables are considered to be independent, and density functions from the exponential family are used [22, 23].

$$f(y_{ij}|\theta_{ij},\phi )=\exp[\phi^{-1}\{y_{ij}\theta_{ij}-\varphi (\theta_{ij})\}+k(y_{ij},\varphi )].$$

where φ is a scale parameter, k(.)is a function solely dependent on y_ij_ and φ, and ψ is a function fulfilling $E(y_{ij}|u_{i})=\varphi^{'}(\theta_{ij})=v(x^{t}{}_{ij}\beta +z^{t}{}_{ij}u_{i})$ and $\mathrm{var}(y_{ij}|u_{i})=\theta \varphi^{''}(\theta_{ij})$, for where v(.) is a known link function, x_ij_ and z_ij_ are covariate vectors and β is a vector of unknown fixed effect parameters.

As a result, this study examined the effect of women and household variables on unintended pregnancy in Ethiopia using the generalized linear mixed model (GLMM) [24–26]. The GLMM model that has been adopted is as follows:

$$h(\mu_{ij})=logit(\mu_{ij})=log\left(\frac{\mu_{ij}}{1-\mu_{ij}}\right)$$

$$h(\mu_{ij})=log\left(\frac{p(y_{ij}=1)}{p(y_{ij}=0)}\right)=\tau_{ij}$$

Where, $\tau_{ij}{=\beta }_{0}+\beta_{1}x_{1ij}+\beta_{2}x_{2ij}+\ldots {+\beta }_{k}x_{kij}+u_{0j}$, X_1ij_.The k explanatory variables measured on women are denoted by X_kij_. The probabilities of women having an unwanted pregnancy and not having an unintended pregnancy are μ_ij_ and 1-μ_ij_respectively (j = 1,…,72 Zones, i = 1,…,n_j_ women within each zone). β_0_ is the log odds of intercept, β_1_…β_k_ are women’s impact sizes of women and u_0j_ are random errors at the Zone level. The distribution of the error term is $u_{0j}∼N(0,{\delta^{2}}_{u0})$.

Between Zone variance and within Zone variance were used to calculate the intra-class correlation (ICC) as $ICC=\frac{\delta_{u}^{2}}{\delta_{u+}^{2}+\delta_{e}^{2}}$. By solving an extended form of mixed equations, the mixed model technique allows for the estimation of fixed effects using the Best Linear Unbiased Estimation (BLUE) method and the prediction of random effects using the Best Linear Unbiased Prediction (BLUP) method [24, 27]. The value of random effects has been recognized by the BLUP [28, 29], which provides an unbiased method by correcting known sources of variation [30]. The crude and BLUP estimations of zones were integrated with the country’s shape data to demonstrate the performance of each zone utilizing maps in this study. Modeling and assessing predictors of unintended pregnancy were done with STATA software, while spatial distribution and mapping were done with ArcGIS version 10.1.

### Ethical approval

Ethical clearance was obtained from measure EDHS by requesting for accessing data. After authorization was allowed through a web ask by clarifying the objective of our investigation, the information was gotten from the DHS program official database, www.meauredhs.com. The data used in this study are publicly available, aggregated secondary data with no personal identifying information that can be linked to study participants and accessed the data set from the DHS website (http://dhsprogram.com) through registering. Since we used a secondary publicly available data set, ethical approval was not required.

## Results

### Study participants characteristics

In this study, 1,0731 pregnant women between the ages of 15 and 49 were enrolled. Among the unintended pregnancies, more than half (50.5%) have occurred in the age group of 25–34 years. In terms of place of residence, 144 (68.6%) of unintended pregnancies occurred in rural areas. Muslim women made up the majority of the unintended participants, accounting for 44.3%Similarly, 116 unintended participants (55.29%) were illiterate, and women with a low-income index (poorer and poorest) accounted for 49.3%of unintended births (Table 1).

**Table 1:** Background characteristics of Ethiopian research participants from the 2016.

Current pregnancy		
**Variables** Intended, n (%)	Unintended, n (%)	
Age		
15–24	342(39.6)	61(29.1)
25–34	416(48.2)	106(50.5)
35+	105(12.2)	43(20.4)
Religion		
Orthodox	217(25.1)	63(30)
Muslim	500(57.9)	93(44.3)
Others	146(17)	54(25.7)
Residency		
Urban	245(28.4)	66(31.4)
Rural	618(71.6)	144(68.6)
Education for Women		
No education	484(56.1)	116(55.29)
Primary	249(28.9)	71(33.81)
Secondary and Higher	130(15.0)	23(10.9)
Index of Wealth		
Poorest	320(37.1)	56 (26.7)
Poorer	130(15.1)	47(22.4)
Middle	116(13.4)	32(15.2)
Richer	91(10.5)	35(16.7)
Richest	206(23.9)	40(19)
Prevalence of unintended pregnancy	29.49% [95%; CI: 23.52%, 34.45%)	

### Prevalence of unintended pregnancy

**Fig 3:**
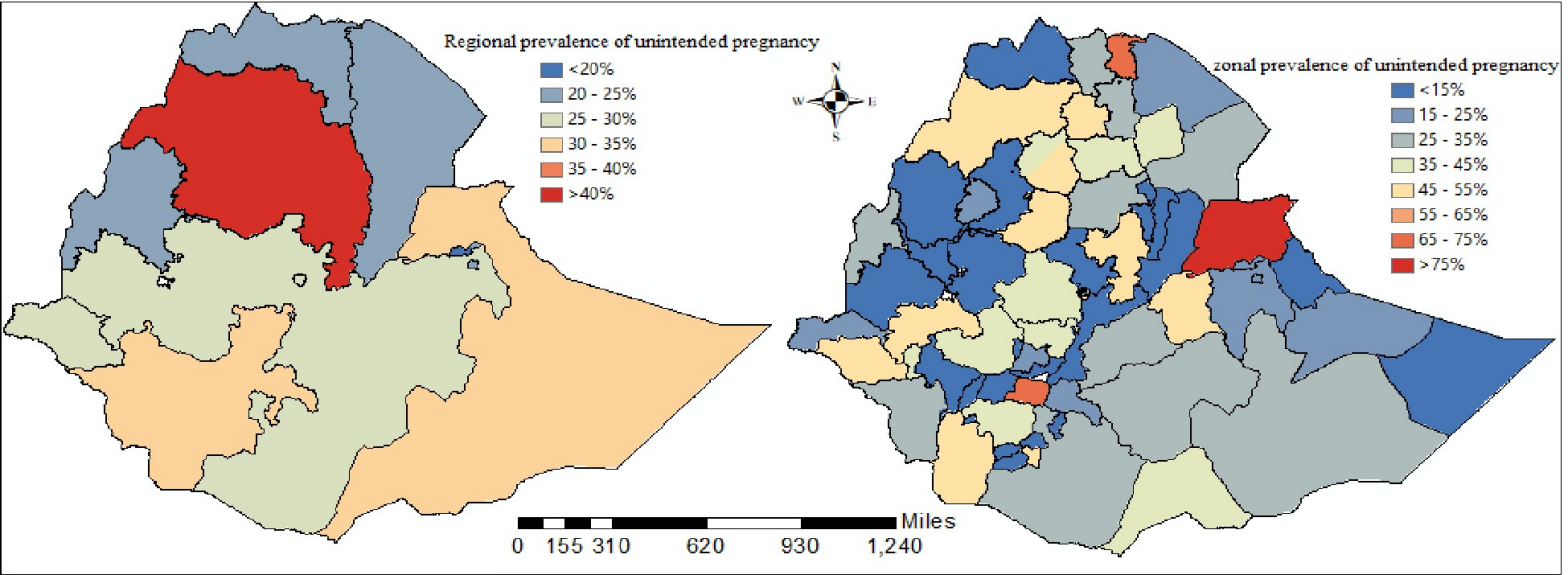
Regional and Zonal, distribution of unintended pregnancy in Ethiopia.

In Ethiopia, the total prevalence of unintended pregnancy was 29.49%[95%; CI: 23.52%, 34.45%] among reproductive women. Amhara and Benshagul Gumuz regions had the highest prevalence of unintended pregnancy, while the Afar and Oromiya regions had the lowest frequency. The zonal prevalence of unintended pregnancy was highest in Dire Dawa administrative city, Shiniel zone, and Hundene, while it was lowest in Dawuro wolayta zone, Amaro Special Woreda, and Benchi Maji zone (Fig 3).

The zones were ranked based on the BLUP estimates, and the top "best" five with the lowest BLUP value and the top five with the highest top "worst" BLUP values performing zones were selected, and thus; Wolayita and Guraghe zones in SNNP, South Gondar and North Gondar zones in Amhara region, and Jimma in Oromiya region were the top "worst" performing zones in terms of unintended pregnancies (Fig 3).

### Spatial distribution of unintended pregnancy

**Fig 4:**
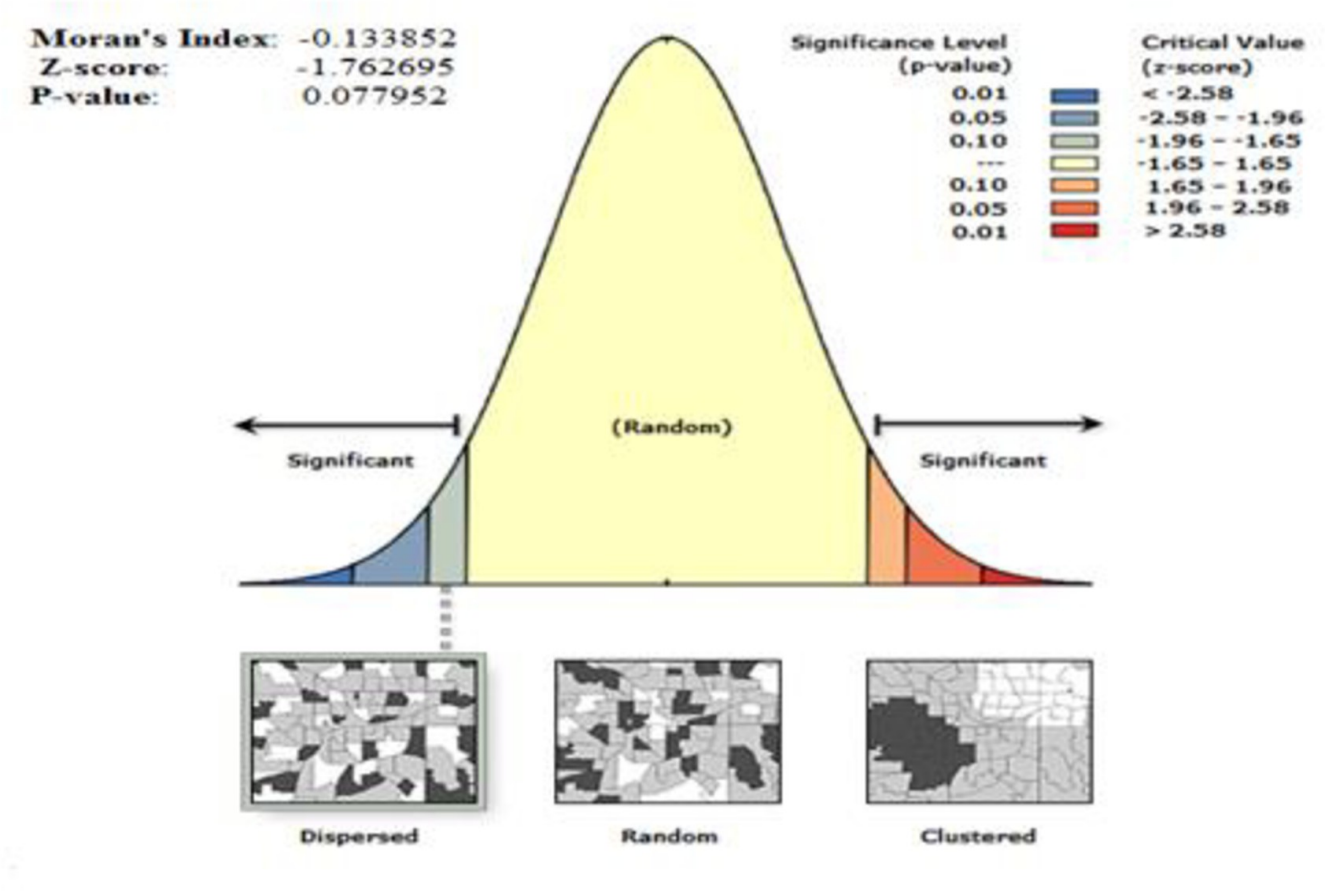
Spatial autocorrelation analysis of unintended pregnancy among reproductive women in Ethiopia, 2016.

The spatial distribution of unintended pregnancies among reproductive women was not uniform in Ethiopia. All over the country, unintended pregnancy is spatially clustered with Global Moran’s I value of -0.134 (p < 0.1) given the z-score of -1.76, there is a less than 10% likelihood that this dispersed pattern could be the result of random chance (Fig 4).

**Fig 5:**
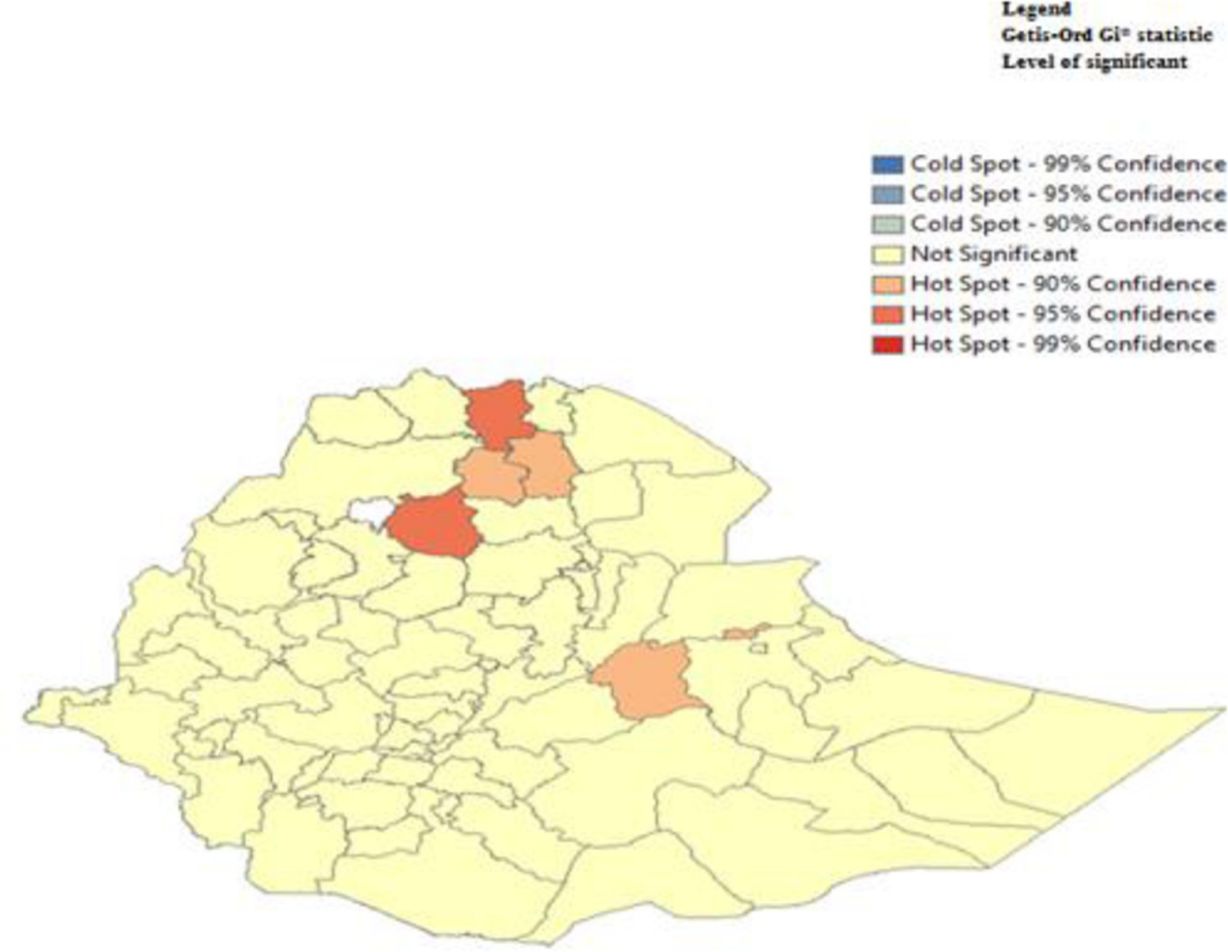
Hotspot and cold spot areas of unintended pregnancy among reproductive age women in Ethiopia.

From the hot spot and cold spot analysis, the red color indicates that the hotspot areas for the prevalence of unintended pregnancy were found in south Gondar, central Tigray, West Harargie, Dire Dawa, Wagemihira and western Tigray zones while the cold spot (low) prevalence was found in West Shewa zone (Fig 5).

### Factors associated with unintended pregnancy

In the multivariable mixed-effect survey logistic regression analysis approach, variables with a significant level of P <0.25 were incorporated into the model to look at the effects of the linked factors on unintended pregnancy. Unintended pregnancy was linked to age, working status, number of children, domicile, wealth index, marital status, women’s education, religion, alcohol consumption, and chat chewing, as shown in Table 2.

**Table 2:** Mixed-effects logistic regression analysis of factors associated with unintended pregnancy among reproductive women in Ethiopia, 2016 (*N* = 1073).

Variables	COR (95% CI)	p-value	AOR (95% CI)	p-value
**Age (**Ref. = 15–24 year**)**				
15–24	1		1	
25–34	1.29(0.76, 2.16)	0.337	0.78(0.62, 0.97)	0.032**
>35	1.74(1.01, 2.98)	0.044*	0.15(1.06, 1.36)	0.007**
**Working Status** (Ref. = Yes)				
No	1		1	
Yes	1.64(1.10, 2.44)	0.015*	1.85(1.14, 2.98)	0.012**
**No of under 5-year children** (Ref = 0)				
0	1		1	
1	2.02(1.17, 3.48)	0.012*	2.93(1.53, 5.59)	0.001**
2	4.94(2.60, 9.37)	0.000*	3.45(3.35, 5.72)	0.000**
> = 3	2.63(0.85, 8.10)	0.092*	4.106(1.17, 1.48)	0.028
**Residency (**Ref. = Urban**)**				
Urban	1		1	
Rural	2.18(1.17, 4.07)	0.014*	2.62(1.94, 7.27)	0.040**
**Index of Wealth (**Ref. = Poorest**)**				
Poorest	1		1	
Poorer	2.51(1.57, 2.12)	0.170*	1.89(1.51, 2.31)	0.033**
Middle	1.47(1.19, 2.07)	0.092	1.75(1.43, 3.12)	0.001**
Richer	1.35(1.67, 2.65)	0.773	1.56(1.54, 2.91)	0.007**
Richest	1.04(0.02, 1.23)	0.674	1.37(0.45, 4.15)	0.581
**Marital Status of women (**Ref. = Other**)**				
Others	1		1	
Married	0.04(0.01, .24)	0.000*	0.05(0.01, 0.38)	0.004**
**EDU status of husband (**Ref. = non-Educated**)**				
Non-Educated	1		1	
Primary	1.27(0.81, 196	0.307 .	1.21(0.67, 2.19)	0.522
Secondary & Higher	0.59(0.31, 1.07)	0.083*	0.87(0.37, 2.12)	0.785
**EDU status of Women** (Ref. = non-Educated)				
Non-Educated	1		1	
Primary	0.97(0.64, 1.47)	0.876	1.34(0.75, 2.39)	0.316
Secondary & Higher	0.55(0.27, 1.09)	0.089*	2.14(1.79, 5.76)	0.031**
**Religion** (Ref. = Orthodox)				
Orthodox	1		1	
Muslim	1.21(1.72, 2.02)	0.046*	0.84(0.79, 1.76)	0.007**
Others	1.12(0.66, 1.89)	0.665	0.92(0.41, 2.08)	0.832
**Media Access** (Ref. = No)				
No	1		1	
Yes	0.70(0.47, 1.06)	0.089*	0.92(0.55, 1.53)	0.749
**Chawing of khat** (Ref. = No)				
No			1	
Yes	1		1.58(0.78, 3.17)	0.202
**Alcohol Use** (Ref. = No)	1.55(0.92, 2.61)	0.099*		
No	1		1	
Yes	1.89(1.57,2.04) *	0.183*	1.09(1.49, 2.42)	0.032**
**Smoking Cigarettes** (Ref. = No)				
No	1		1	
Yes	2.43(1.00, 5.92)	0.049*	3.67(1.17, 11.56)	0.026**
**Random-effect Parameter**		**ICC**	**Variance (u**_**i**_ **)**	**p-value**
Zone		0.09	0.036	0.000**

COR = crude odds ratio, AOR = adjusted odds ratio, I CC = Intra class correlation, EDU = Education.

*Significant (p<0.25)

**significant (p<0.05).

Women aged 25–34 and > = 35 were 22% [AOR = 0.78, 95%CI, 0.62–0.97] and 85% [AOR = 0.15%, 95%CI, 1.06–1.36] less likely to have an unintended pregnancy than women aged 15–25 years. Women with 1 and 2 children were 2.93 times to have an unintended pregnancy [AOR = 2.93, 95%CI, 1.53–5.59] and 7.45 times to have an unintended pregnancy [AOR = 7.45, 95%CI, 3.53–15 .72] compared to women who had never had a child.

Muslim women were 0.84 times less likely than orthodox women to be unintended [AOR = 0.84, 95%CI, 0.79–1.76]. Women who chew khat and drink alcohol had an unintended pregnancy 1.6 times [AOR = 1.58, 95%CI, 0.78–3.17] and 1.1 times [AOR = 1.09, 95%CI, 1.49–2.42] than women who did not chew khat and did not drink alcohol respectively.

When compared to the poorest wealth index, women with a poorer, middle, richer, and richest wealth index were 1. 9 times [AO = 1.89, 95%CI%, 1.51–2.31], 1.75 times [AOR = 1.75, 95% CI, 1.43–3.12], and 1.56 times [AOR = 1.56, 95%CI, 1.54, 2.91] to have an unintended pregnancy respectively. The Intra—Class Correlation (ICC) of the mixed model was 0.09, which is quite tiny, implying that there was a substantial variation in unintended pregnancy across the zones.

## Discussion

According to the study, Ethiopia’s country-wide prevalence of unintended pregnancy was around 29.49%. The finding of this study was similar with the studies in Ethiopia [31, 32] but higher than the results of the studies conducted in Ethiopia [33–35]. The reason for this difference may be that this study includes women aged 15–49 years while other studies [33, 35, 36] only used youth women (15–24 years).This prevalence was higher than the finding conducted in Ethiopia [35] but lower than in Ghana [36], Malawi [37], and the Democratic Republic of Congo [38]. The differences might be due to differences in sexual and reproductive health service coverage and study period. Another possible reason is that in Ethiopia, health extension workers are assigned to community services through home visits, so awareness of intended pregnancy may be higher than in other African countries such as Kenya and Ghana, and there may also be differences in health coverage across African countries. The findings could possibly be lower due to a tendency to report a pregnancy as intended once it has occurred, as well as a difference in sample design, as we used complicated weighted sampling in this study.

The results of the spatial analysis revealed that there is variation in exposure to unintended pregnancy among reproductive women across the both regional and zonal states of Ethiopia. A high prevalence of unintended pregnancy was found in south Gondar, central Tigray, west Harargie, Dire Dawa, Wagemihira and western Tigray zones of Ethiopia. This result is consistent with the findingsby Kebede and his colleagues [35]. The prevalence of unintended pregnancy was higher in most zones of the Amhara, SNNPR, and Oromiya regions, while the lowest prevalence was found in Tigray regional zones. This might be due to cultural differences and religious differences among women along the lines of region and zone. Furthermore, inequality in contraceptive use and quality of family planning counseling across the region as well as the zone might be the reason for this variation.

The mixed-effect survey logistic regression analysis revealed that older women were shown to be strongly related to unintended pregnancy in this study, which is similar to the findings in different studies [7, 8]. This implies that older women have a better understanding of how to use contraception to avoid unintended pregnancy. Moreover, women aged 15–24 years were shown to be more likely than women of other ages to have an unintended pregnancy. This conclusion is consistent with the findings from Pakistani [39]. Younger women are more fertile, have more sexual intercourse, are less likely to seek family planning assistance, have more contraceptive failure than older women, and have more misunderstanding about contraceptive usage, all of which lead to a higher risk of unintended pregnancies [39–41].

Our finding also revealed that the woman’s educational level has a substantial impact on unintended pregnancy. This research demonstrated a negative association between the chance of unintended pregnancy and a pregnant woman’s educational degree. As a result, highly educated women have a far lower probability of becoming pregnant unintentionally. This result is consistent with the findings by Indian and Malawi [37, 42]. This implies that women with a higher level of education are more empowered to control their sexual and reproductive health than women with little formal education.

Unintended pregnancy is substantially associated with more children, in line earlier studies conducted in Ethiopia [9, 43] and Nigeria [44], with women having three or more children having a higher risk of unintended pregnancy. This could be because women with several children are preoccupied with caring for them, which hinders their access to knowledge and the use of contraceptive techniques, resulting in unintended pregnancy.

Moreover, religion is the single most important factor linked to unintended pregnancies. In comparison to orthodox women, Muslim mothers were less likely to have an unintended pregnancy. This finding is consistent with a research conducted in Ghana [45] and Ethiopia [46]. Women who drank alcohol and chewed chat were also more likely to have unexpected pregnancies. This implies that using alcohol or chat had positive contribution of unintended pregnancy which is in line with the studies conducted in Ethiopia [47, 48], this may be due to the failure to employ a contraceptive method. Furthermore, it was discovered that when one’s wealth position rises, the likelihood of an unintended pregnancy decreases. Wealthier women, unlike the impoverished, are less likely to have unintended pregnancies. This finding is comparable to those found in studies conducted in Iran [43] and Kenya [39].

This study has a number of strengths. The first strength of the study was that it used a large sample size data from the typical Ethiopian Demographic and Health Survey. Secondly, the study identified as hotspot locations of unintended pregnancy, this work contributes to the corpus of knowledge on the spatial disparity of unintended pregnancy in Ethiopia. Lastly, for a more full and accurate analysis, the study employs both geographical and non-spatial statistical methodologies. This study has a number of limitations. One of the limitations of this study is that data was froma secondary source and the survey used a cross-sectional design to collect data as such no pivotal extrapolations can be made. In addition, the 2016 EDHS survey participants self-reported their pregnancies. Women reported that their pregnancies were intended, though it is possible that some pregnancies were not actually intended. This could underestimate the burden of unintended pregnancies.

## Conclusion

This study found that there are spatial clusters of unintended pregnancies among reproductive age in Ethiopia. The prevalence of unintended pregnancy is high among reproductive women. Both the crude prevalence and the BLUP demonstrate that there was a lot of diversity in unintended pregnancy in Ethiopia at the zonal level. Unintended pregnancy was also linked to age, residential wealth index, religion, women’s education, marital status, working status, and the number of children under the age of five. Furthermore, the rural residence, particularly the Amhara, SNNPR, and Oromiya regional zones, has a high proportion of unintended pregnancy. Hence, we recommended creating awareness of sexual and reproductive health with special priority to the identified hotspot areas (Amhara, Oromiya and SNN regions) to reduce unintended pregnancy. Emphasis on fertility and contraceptive techniques should be given to couples. The improvement of the delivery of efficient information, education, and counseling about unintended pregnancy is urged upon policy makers, health practitioners, and health authorities.
